# NMR Structure, Dynamics and Interactions of the Integrin β2 Cytoplasmic Tail with Filamin Domain IgFLNa21

**DOI:** 10.1038/s41598-018-23866-6

**Published:** 2018-04-03

**Authors:** Deepak Chatterjee, Lewis Lu Zhiping, Suet-Mien Tan, Surajit Bhattacharjya

**Affiliations:** School of Biological Sciences, Nanyang Technological University, 60 Nanyang Drive, Singapore, 637551 Singapore

## Abstract

Integrins are transmembrane proteins that mediate cell adhesion and migration. Each integrin is a heterodimer formed by an α and a β subunit. A large number of cytoplasmic proteins interact with the cytoplasmic tails (CTs) of integrins. The actin-binding cytoskeletal protein filamin A is a negative regulator of integrin activation. The IgFLNa21 domain of filamin A binds to the C-terminus of β2 CT that contains a TTT-motif. Based on x-ray crystallography, it has been reported that the integrin β2 CT forms a β strand that docks into the β strands C and D of IgFLNa21. In this study, we performed solution NMR analyses of IgFLNa21 in the presence of integrin β2 CT peptides, and hybrid IgFLNa21, a construct of covalently linked IgFLNa21 and β2 CT. The atomic resolution structure of the hybrid IgFLNa21 demonstrated conserved binding mode with β2 CT. Although, ^15^N relaxation, model free analyses and H-D exchange studies have uncovered important insights into the conformational dynamics and stability of β2 CT in complex with IgFLNa21. Such dynamical characteristics are likely to be necessary for the TTT-motif to serve as a phosphorylation switch that regulates filamin A binding to integrin β2 CT.

## Introduction

Integrins are heterodimeric transmembrane receptors that mediate cell-cell adhesion and cell-anchorage to extracellular matrix^1^. In addition, they transmit bi-directional signals by undergoing conformational changes^2^. The conversion of integrin mechanical signals to cellular biochemical signals and vice versa are crucial for anchorage-dependent cells to sense and respond to their local environment^3^. The short cytoplasmic tails (CTs) of most integrins interact with a large number of cytoplasmic proteins that serve either as positive or negative regulators of integrin activation and outside-in signalling^4^. Many of these regulators are high large proteins and they have overlapping binding sites in the integrin CTs^4^. Conceivably, competition for binding to the integrin CTs by these molecules is an important mechanism in the regulation of integrin function.

The CTs of integrin β subunits are highly conserved^5^. Notably, two NxxY/F motifs, one membrane proximal and the other membrane distal, have been shown to bind talin and kindlins, respectively^6–8^. Talin and kindlins are well established positive regulators of integrin activation^9^. On the other hand, filamin A, which has a binding interface that overlaps with that of talin and kindlins, has been shown to be a negative regulator of integrin β2 and β7^10–12^. Mechanistically, an increase in association of filamin A with the integrin β CTs precludes talin binding as a result of steric hindrance. To facilitate integrin activation, kindlins in association with migfilin has been suggested to displace filamin A from the integrin cytoplasmic tail, thereby favoring talin-integrin tail interactions^13,14^. Phosphorylation of Thr758 in the integrin β2 CT has also been shown to disrupt filamin A binding^15,16^.

Filamins are a family of three actin-binding proteins (FLN A,B,C)^17^. Filamin A consists of two ~280 kDa monomers that are linked at their C-termini, which give rise to a V-shaped conformation suffice for the branching of actin filaments. A key feature of the filamin monomer is the presence of 24 Ig-like β-sheet repeats (IgFLNa1-24). It has been shown that IgFLNa21 binds to the CTs of integrins β2 and β7 with the latter forming a β strand that hydrogen bonds with and runs anti-parallel to the IgFLNa21 βC strand^11,12^. In both integrin β2 and β7 CTs, a motif containing three Thr is found between the two NPxF motifs^5^. The T(758)TT-motif in integrin β2 CT is amenable for phosphorylation^15,16^, and it has been shown that phosphorylation of Thr758 diminished IgFLNa21 binding^12^. To date, molecular insights into IgFLNa21 and integrin β2 and β7 interactions are largely derived from x-ray crystallography studies^11,12^. Notably, dimeric structure was observed for IgFLNa21 in complex with β7 CT whereas a monomeric complex was determined with β2 CT^11,12^. More recently, a structure of IgFLNa21 has been reported in a ternary complex of β3 and αIIb CTs of platelet integrin by NMR spectroscopy^17^. In this study, using solution NMR, we investigated interactions of IgFLNa21 with integrin β2 CT and determined 3-D structure of a covalent complex of IgFLNa21/β2 CT. Backbone ^15^N relaxation parameters and H-D exchange rates were compared for hybrid IgFLNa21 and IgFLNa21. Our results provide important molecular insights toward filamin mediated regulation of integrins.

## Results

### Interactions of IgFLNa21 with integrin β2 CT by ^15^N-^1^H HSQC and NMR studies of β2 CT conjugated IgFLNa21 filamin

**Figure 1:**
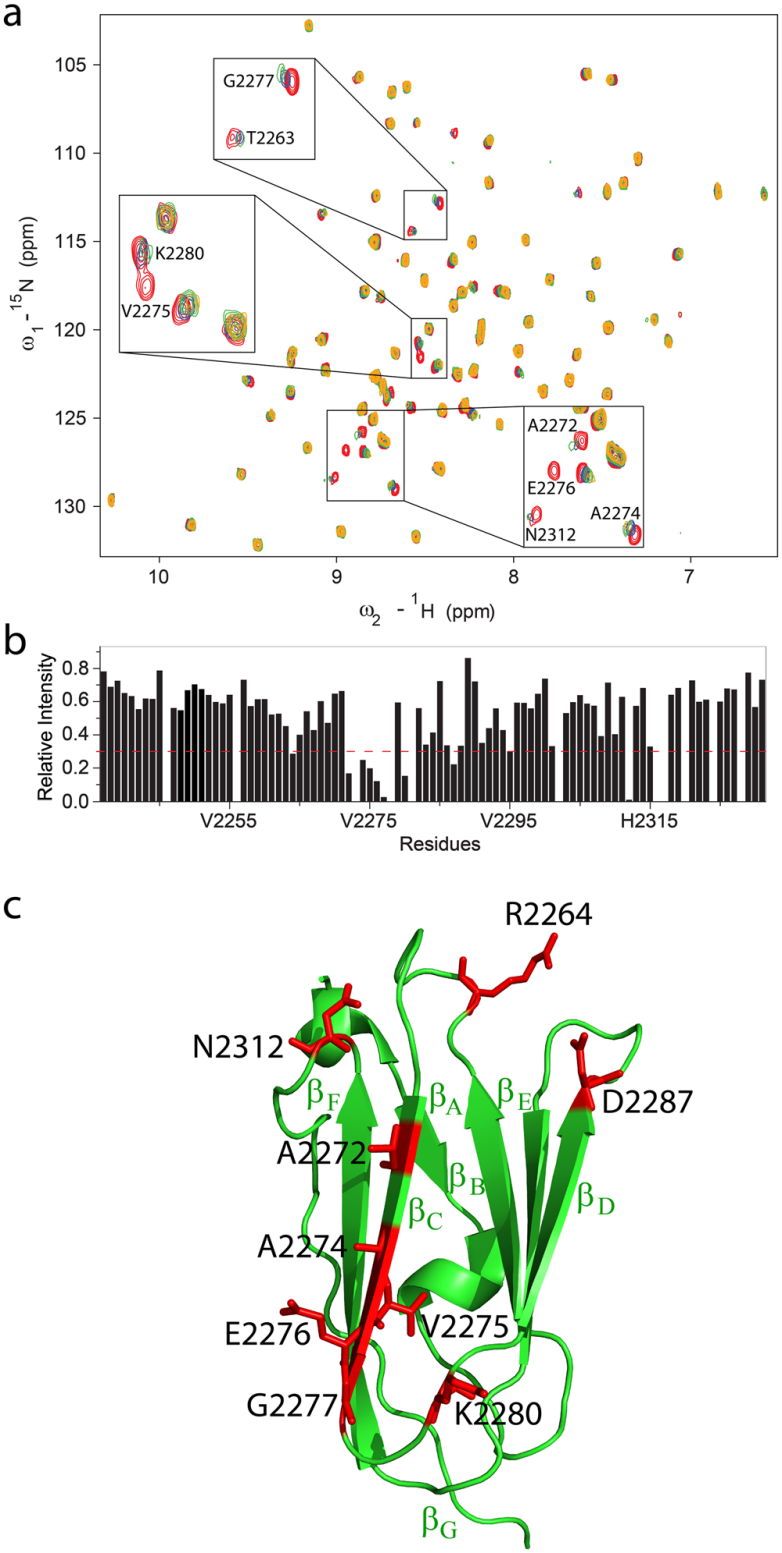
Interactions and mapping residues of IgFLNa21 with β2 CT. (**a**) Overlay of ^15^N-^1^H HSQC spectra of IgFLNa21 in free solution (in yellow) and in presence of β2 CT at 1:1 (in red) and in 1:3 (in light green) molar ratios. (**b**) Bar diagram showing relative intensity changes of ^15^N-^1^H HSQC cross-peaks of IgFLNa21 upon addition of three fold excess of β2 CT. The red line indicates average value of the intensity change. (**c**) A ribbon representation of the 3-D structure of IgFLNa21 (pdb: 2brq). Residues of IgFLNa21 experienced above average intensity changes upon addition of β2 CT are highlighted in red color.

**Figure 2:**
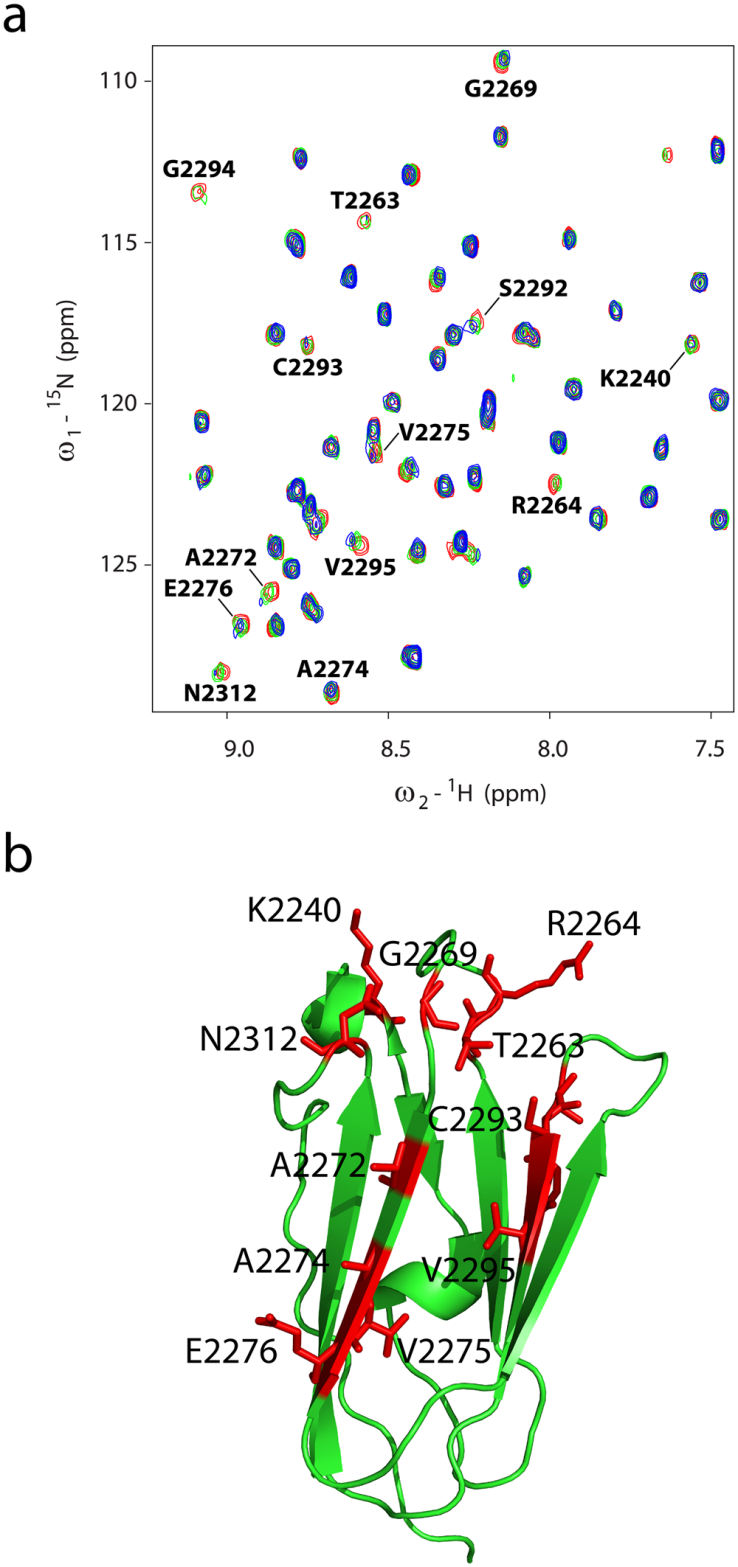
Interactions and mapping residues of IgFLNa21 with β2 CT MP. (**a**) Overlay of ^15^N-^1^H HSQC spectra of IgFLNa21 in free solution (in red) and in presence of β2 CT MP at 1:1 (in green) and in 1:3 (in blue) molar ratios. (**b**) A ribbon representation of the 3-D structure of IgFLNa21 (pdb: 2brq). Residues of IgFLNa21 experienced resonance perturbation upon addition of three fold excess of β2 CT are highlighted in red color.

**Figure 3:**
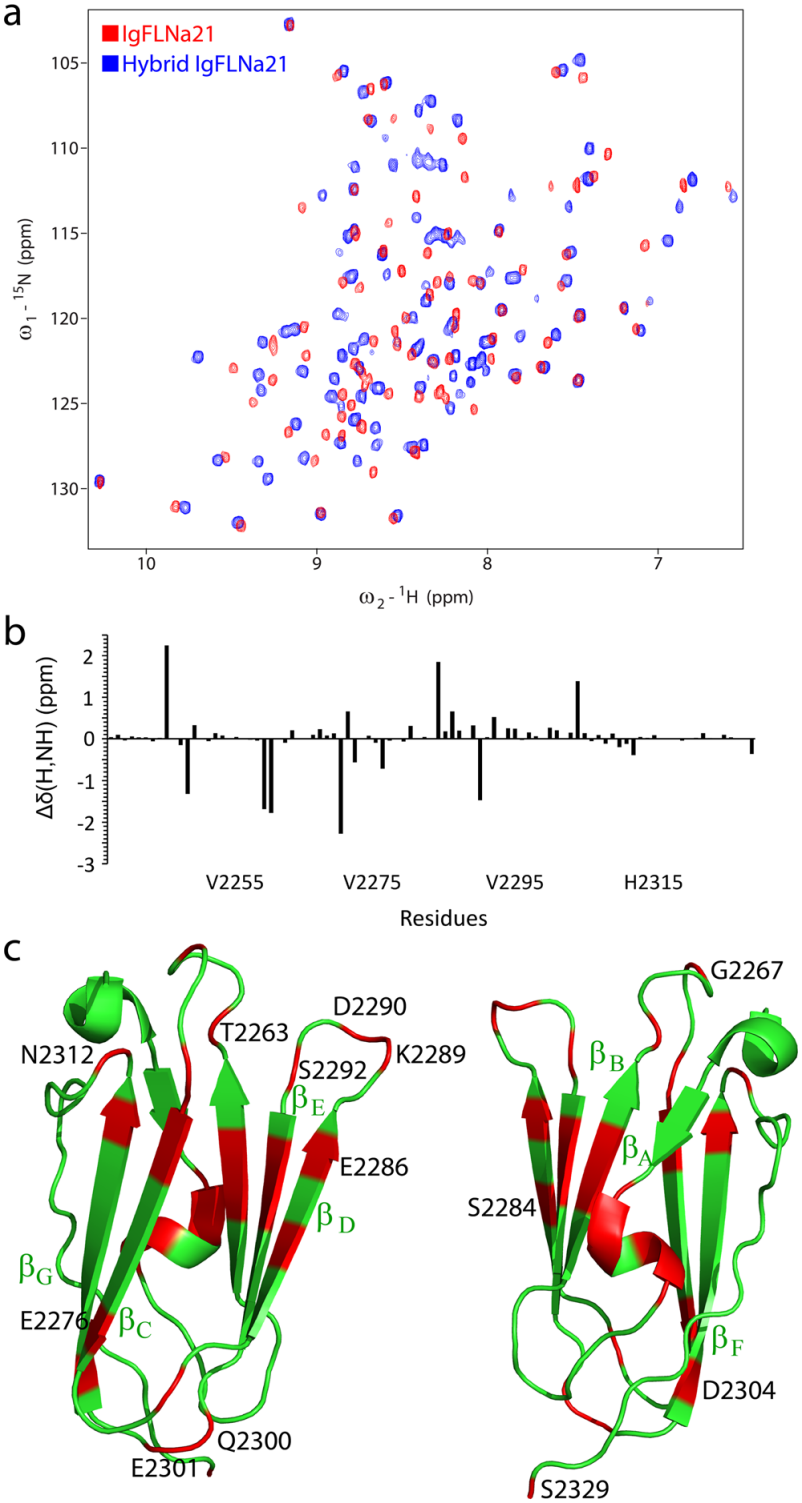
NMR of hybrid IgFLNa21 and chemical shift mapping. (**a**) Overlay of ^15^N-^1^H HSQC spectra of IgFLNa21 (in red) and hybrid filamin (in blue) showing chemical shift differences and folding characteristics. (**b**) Bar diagram showing combined chemical shift differences of ^1^HN and ^15^N resonances between IgFLNa21 and hybrid IgFLNa21 for individual residues of IgFLNa21. (**c**) Ribbon representation of the 3-D structure of IgFLNa21 (pdb: 2brq) in two different orientations. Residues showing combined chemical shift differences >0.2 ppm are highlighted in red.

We first examined the feasibility of obtaining a stable complex of IgFLNa21 with full length integrin β2 CT. The ^15^N-^1^H HSQC spectra of IgFLNa21 in free solution and in the presence of β2 CT at 1:1 and 1:3 ratios are shown (Fig. 1a). The HSCQ spectrum of IgFLNa21 was assigned using triple resonance HNCACB and CBCA(CO)NH experiments. Additions of β2 CT have caused conspicuous changes in the HSQC spectra of IgFLNa21. A number of HSQC peaks were either broadened or disappeared which is typically observed for fast exchanging complexes undergoing transient interactions^18^. A significantly low binding affinity, K_d_ ~ 0.5 mM, was estimated, using surface plasmin resonance method, of β2 CT/IgFLNa21 complex^12^. Regardless, intensity change of HSQC cross-peaks of individual residues of IgFLNa21 were estimated (Fig. 1b) and mapped onto the structure of IgFLNa21 (Fig. 1c). Residues e.g. Ala2272, Ala2274, Val2275, Glu2276 and Gly2277, located at the β-strand C (β_C_) in the ligand binding pocket of IgFLNa21 demonstrated marked perturbations upon binding to β2 CT. In addition, some distal residues, e.g. Arg2264, Asp2287, Lys2280, Asn2312, of IgFLNa21 also experienced binding induced perturbation. Most of the recent reports demonstrated that the C-terminus or membrane distal region of β-CTs bind to the IgFLNa21domain of filamin^11,12,17^. However, a previous study has indicated binding of filamin to the membrane proximal (MP) region of β2 CT^19^. We further investigated interactions of IgFLNa21 with a MP peptide fragment of β2 CT (K^724^ALIHLSDLREYRRFEKEKLKSQWNND^750^). Figure 2a shows overlay of ^15^N-^1^H HSQC spectra of IgFLNa21 at 1:0 (red) and 1:1 (green) and 1:3 (blue) ratios of β2 MP. ^15^ N-^1^H HSQC spectra remains largely invariant at 1:1 ratio, however, discernable spectral changes could be observed at 1:3 ratio (Fig. 2a). A number of ^15^N-^1^H HSQC cross peaks were observed to be broadened upon addition of β2 MP peptide (Fig. 2a). Note, ^15^N-^1^H HSQC titration data of IgFLNa21 with full-length β2 CT delineated changes even at 1:1 ratio (Fig. 1a). These observations, therefore, indicated that the β2 MP peptide interacted rather weakly compared to the full length β2 CT. Residues which demonstrated resonance perturbation at 1:3 ratio of β2 MP are mapped onto the structure of IgFLNa21 (Fig. 2b). A set of topologically close proximal residues, located at the loops, Asn2312, Lys2240, Gly2269, Thr 2263 and R2264 of IgFLNa21 demonstrated binding induced perturbation (Fig. 2b). In addition, residues belonging to β_C_ strand, at the canonical ligand binding pocket, and adjacent β-strand appeared to be influenced by interactions with β2 MP (Fig. 2b). Interestingly, the atomic resolution structure of the high-affinity ternary complex IgFLNa21/β3 CT/αIIb CT demonstrated that the MP helix of the β3 CT docks onto these loop residues of IgFLNa21^20^. Although, we were able to map interactions between IgFLNa21, full length β2 CT and β2 MP peptide, however, the transient binding precluded 3-D structure determination of the complex in solution. In order to prevent fast dissociation of the complex, we constructed a hybrid β2CT-IgFLNa21 in which the C-terminal filamin-binding region (P^752^LFKSATTTVMN^763^) of β2 CT was conjugated to the N-terminus of IgFLNa21 filamin by a flexible linker. This strategy was also employed to determine 3-D structures of transient protein complexes, including that of integrin CT and its binding proteins^20,21^. An overlay of the ^15^N-^1^H HSQC spectra of IgFLNa21 filamin and hybrid IgFLNa21 is shown (Fig. 3a). ^15^N-^1^H HSQC peaks of the hybrid IgFLNa21 construct were well resolved without any resonance broadening. The backbone (^13^Cα, ^15^N, ^1^H) and sidechain resonance assignments of hybrid IgFLNa21 construct were achieved by standard triple resonance NMR experiments (Materials and methods). Further, we analyzed the combined chemical shift difference of ^15^N and NH atoms between IgFLNa21 and hybrid IgFLNa21 (Fig. 3b). Residues of IgFLNa21 showing >0.2 ppm chemical shift difference are highlighted (Fig. 3c). Chemical shift difference, ^15^N and NH, has been mostly reflected for several residues of the ligand binding β-strand and also for adjoining β strands and loops of IgFLNa21 (Fig. 3c). These observations suggest correct insertion of the β2 CT into the binding pocket of IgFLNa21.

### Three-dimensional structure of hybrid β2CT-IgFLNa21

**Figure 4:**
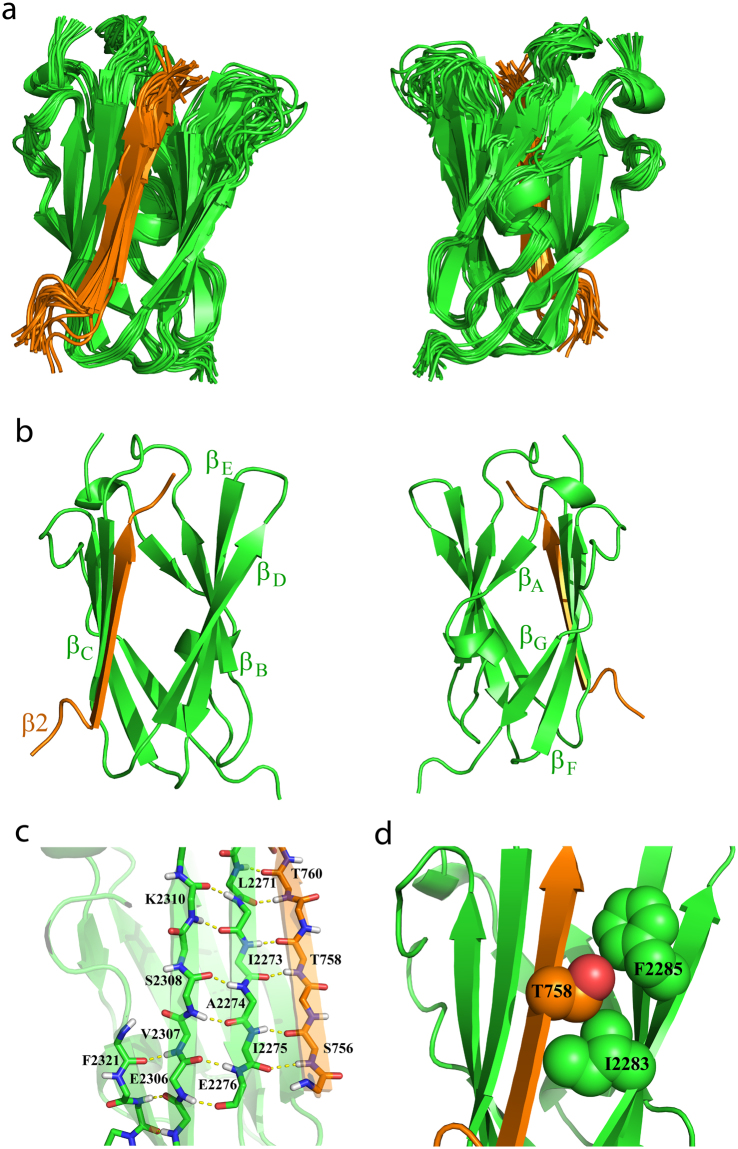
NMR structure of hybrid IgFLNa21. (**a**) Superposition of twenty low energy structures of hybrid IgFLNa21. The structures are shown as ribbon and in two different orientations. The β2 CT part has been shown as orange ribbon. The IgFLNa21 domain is in green. The linker residues are not shown for a better clarity. (**b**) Ribbon representation, in two different orientations, of a selected structure of hybrid IgFLNa21 showing fold and secondary structures. The β2 CT binds to the CD face of IgFLNa21 and assumes an antiparallel orientation with the β strand C of IgFLNa21. (**c**) Potential backbone hydrogen bonding interactions between the β2 CT (in orange) and the β strand C of IgFLNa21 in hybrid IgFLNa21. (**d**) Sidechain-sidechain packing interactions, in space filling representation, involving residue T758 of β2 CT and residues I2283 and F2285 from the β strand D of IgFLNa21 in hybrid IgFLNa21.

Atomic resolution structure of β2CT-IgFLNa21 was determined, by CYANA^22^, and further refined by X-plor-NIH^23^ with explicit water. The superposition of an ensemble of twenty low energy structures of hybrid IgFLNa21 is shown (Fig. 4a). The structural statistics and RMSDs values of the ensemble are listed in Table 1. The NMR structure of hybrid IgFLNa21 revealed seven well-defined β-strands, labeled A to G, whereas the N-terminal residues Gly2236-Pro2256 displayed mixed conformations e.g. short helices and extended β-structures (strand A), possibly due to the occurrence of multiple Gly and Pro (Fig. 4b). The overall topology of the IgFLNa21 domain in hybrid protein is akin to the structures previously determined for filamin repeats including domain 21^11–13,24^. In the hybrid IgFLNa21 structure, the β2 CT peptide adopted a well-defined β-strand that interdigitated into the binding pocket between strands β_C_ and β_D_ of the IgFLNa21domain in a canonical fashion. The β-strand of β2 CT orients anti-parallel to β_C_ and parallel to β_D_, forming a triple stranded β-sheet (Fig. 4b). The atomic resolution structure of hybrid IgFLNa21 reveals that the binding of β2 CT peptide is maintained by an array of backbone-backbone hydrogen bonds between residues in the β-strand of β2 CT and residues in the β_C_ strand of the IgFLNa21 domain (Fig. 4c). On the other hand, side chain-side chain packing interactions are predominant between β2 CT and the strand β_D_ of IgFLNa21 domain (Fig. 4d). In particular, alkyl side chain of residue Ile2283 and aromatic side chain of residue Phe2285 are closely packed with side chains of Thr758 and Thr760 of β2 CT (Fig. 4d). Notably, the side-chain of the phosphorytable residue Thr758 in the TTT motif of β2 CT is engaged in close packing interactions with both Ile2283 and Phe2285 (Fig. 4d). Calculation of solvent accessible surface area of residues of hybrid IgFLNa21 indicated that Thr758 is buried inside the core of the structure.

**Table 1:** Structural statistics of the ensemble of twenty lowest energy structures of hybrid IgFLNa21.

**NMR distance and dihedral angle constrains**	
Distance constraints	
Short-range (i–j) ≤ 1	355
Medium-range 1 < (i–j) < 5	548
Long-range (i–j) ≥ 5	598
Total	1501
Dihedral angle constraints	
Φ	68
Ψ	68
**Structure statistics**	
Violations (mean + s.d.)	
Distance constraints (Å)	0.072 ± 0.002
Dihedral angle constrains (°)	1.390 ± 0.021
Deviations from idealized geometry	
Bond lengths (Å)	0.010 ± 0.000
Bond angles (°)	0.789 ± 0.019
Average pairwise r.m.s. deviation (Å)^a^	
Heavy atoms	0.9
Backbone	0.5

^a^Pairwise r.m.s. deviation was calculated for ordered residues among twenty refined structures.

### ^15^N Relaxation and dynamics of IgFLNa21 and hybrid IgFLNa21

**Figure 5:**
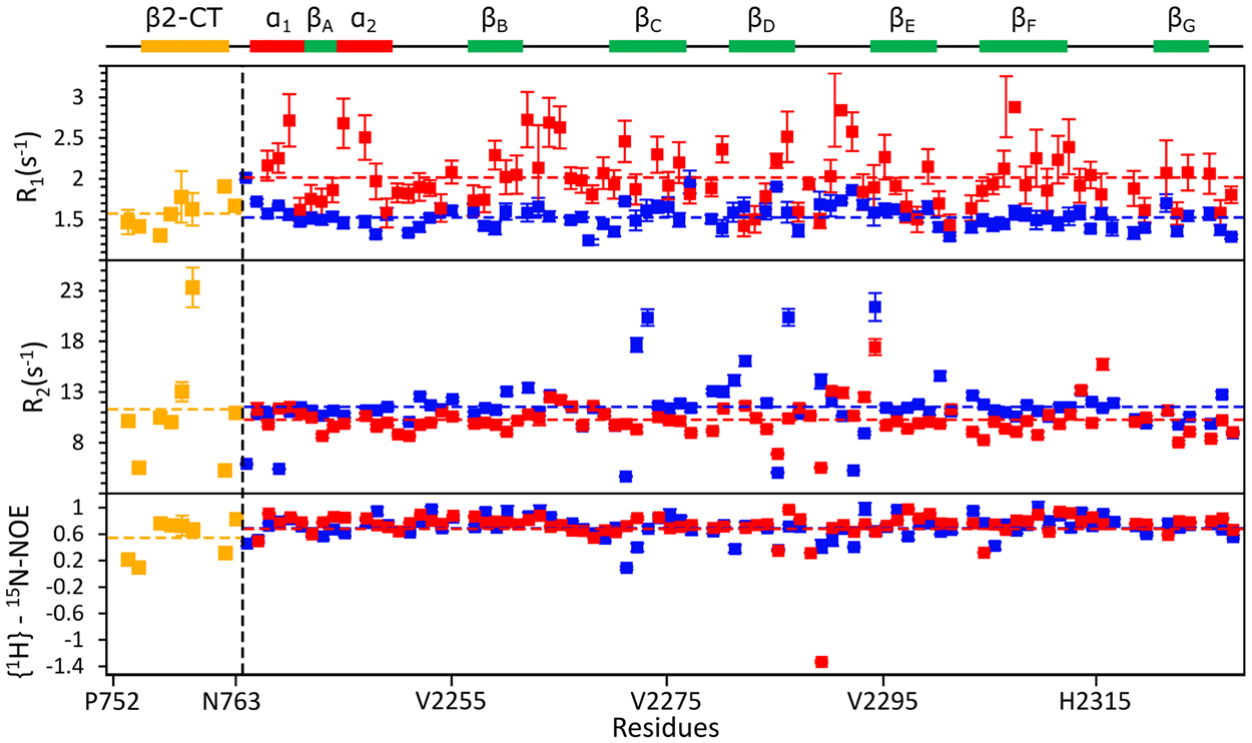
Backbone dynamics of IgFLNa21 and hybrid IgFLNa21. R_1_ (top panel), R_2_ (middle panel) and heteronuclear NOE (bottom panel) of IgFLNa21 and hybrid IgFLNa21. Relaxation data for residues belonging to the domain of IgFLNa21 and hybrid IgFLNa21 are shown in red and blue color, respectively. Relaxation data for residues of the β2 CT part of hybrid IgFLNa21 has been shown in yellow. The boundary between β2 CT and IgFLNa21 domain has been indicated by a vertical broken line. The horizontal broken lines in the plot indicate average value of the relaxation parameters. A schematic representation of the secondary structures of hybrid IgFLNa21 has been shown at the top.

**Figure 6:**
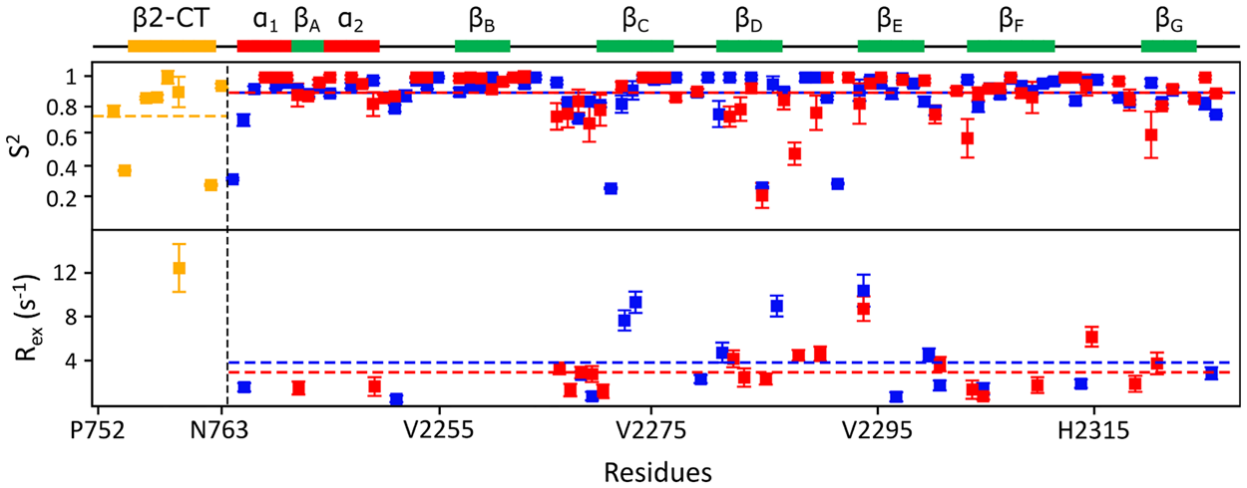
Model free analyses of IgFLNa21 and hybrid IgFLNa21. Generalized order parameter or S^2^ (top panel) and R_ex_ (bottom panel) values of residues of IgFLNa21 and hybrid IgFLNa21. S^2^ and R_ex_ values for residues belonging to the domain of IgFLNa21 and hybrid IgFLNa21 are shown in red and blue color, respectively. S^2^ and R_ex_ values for residues of the β2 CT part of hybrid IgFLNa21 have been shown in yellow. The boundary between β2 CT and IgFLNa21 domain has been indicated by a vertical broken line. The horizontal broken lines in the plot indicate average value of the relaxation parameters. A schematic representation of the secondary structures of hybrid IgFLNa21 has been shown at the top.

Backbone ^15^N spin relaxation parameters, R_1_, R_2_ and hetero-nuclear NOE of both IgFLNa21 and hybrid IgFLNa21 were estimated (Fig. 5). These relaxation parameters are sensitive to fast motion of the N-H bond vector at pico-second and nano-second time scale. Relaxation parameters can be obtained for non-overlapping resonances of 88 and 81 non prolyl residues out of 97 and 86 non prolyl residues of hybrid IgFLNa21 and IgFLNa21, respectively. Backbone dynamics of IgFLNa21 and hybrid IgFLNa21 were further analyzed by use of model free formalism. The R_1_ values of residue of hybrid IgFLNa21 were uniform with less variation across the sequence (Fig. 5, top panel). The average R_1_ values for the β2 CT and IgFLNa21 domain in hybrid IgFLNa21 were estimated to be 1.59 and 1.55 s^−1^, respectively. Whereas the N-terminal residues including residues in strands β_A_ and β_B_ of IgFLNa21 showed somewhat higher R_1_ values compared to hybrid IgFLNa21 with an average R_1_ value of the domain 2.09 s^−1^ (Fig. 5, top panel). The hybrid IgFLNa21 and IgFLNa21 demonstrated that most of the residues in the IgFLNa21domain have experienced similar R_2_ values with an average of 11.09 and 10.39 s^−1^, respectively, (Fig. 5, middle panel). Further, the residues in the β2 CT of hybrid IgFLNa21 showed an average R_2_ of 11.59 s^−1^. Notably, some residues of the hybrid IgFLNa21 that include residue Thr759 of β2 CT, and residues Ala2272, Ile2273, Glu2286 and Gly2294 belonging to IgFLNa21 domain demonstrated significantly higher R_2_ values. By contrast, only residue Gly2294 of IgFLNa21 displayed higher R_2_ value .The increased R_2_ values suggest potential conformational exchange processes involving these residues of hybrid IgFLNa21. Notably, residues Ala2272, Ile2273, Glu2286 of IgFLNa21 and residue Thr759 of β2 CT are located in the binding sites. Figure 5 (bottom panel) shows heteronuclear NOE of β2CT-IgFLNa21 and wild-type IgFLNa21. Except for some residues in the loop, most of the residues that belong to the IgFLNa21 domain in hybrid IgFLNa21 and IgFLNa21 demonstrated high heteronuclear NOE values with estimated average values of 0.72 and 0.71, respectively (Fig. 5, bottom panel). Such heteronuclear NOE values are indicative of limited motion of N-H bond vectors characteristics of folded proteins^25–27^. The β2 CT residues of β2CT-IgFLNa21, except for terminal residues Leu753 and Phe754, delineated high heteronuclear NOE with an average of 0.71 (Fig. 5, bottom panel). Therefore, analyses of relaxation parameters indicated that the residues of the β2 CT region of hybrid IgFLNa21 have experienced dynamical characteristics akin to the filamin domain. Further, incorporation of the β2 CT into the IgFLNa21 domain yielded dynamical changes for certain residues, as indicated by above average R_2_ values, at the binding pocket. Model free formalism provides quantitative assessment of fast time scale backbone motion^28–30^. The generalized order parameter (S^2^) and R_ex_ were extracted and plotted for each residue of hybrid IgFLNa21 and filamin IgFLNa21 (Fig. 6). The generalized order parameter (S^2^) of the β2 CT in the hybrid IgFLNa21, excluding two terminal residues, demonstrated an average value of 0.81 ± 0.03, indicating restricted backbone motion at ps-ns time scale. Average S^2^ values of 0.89 ± 0.02 and 0.89 ± 0.03 were estimated for the IgFLNa21 domain of hybrid IgFLNa21 and wild-type IgFLNa21, respectively. The high S^2^ values indicated motional rigidity of the IgFLNa21 domain either in the presence of β2 CT or in isolated domain. Low S^2^ values (<0.4) can be seen for some residues located in the loop between the β_C_ and β_D_ and at the C termini (Fig. 6, top panel). ^15^N conformational exchange contributions (R_ex_) to R_2_ rate constant were observed for 19 and 21 residues for β2CT-IgFLNa21 and IgFLNa21 (Fig. 6, bottom panel). Notably, a single residue, Thr759 of β2 CT demonstrated the highest R_ex_ value in hybrid IgFLNa21. Further, residues Ala2272, Ile2273, Ala2281, Glu2286 of hybrid IgFLNa21, displayed above average R_ex_, are located in the binding pocket of strands β_C_ and β_D_. However, these residues appeared to be not involved in R_ex_ in the wild-type IgFLNa21. Other residues in the hybrid IgFLNa21 and wild-type IgFLNa21 that experienced R_ex_ are located in the loops or edges of the β-strands.

### H-D exchange of backbone amide protons of IgFLNa21 and hybrid IgFLNa21

**Figure 7:**
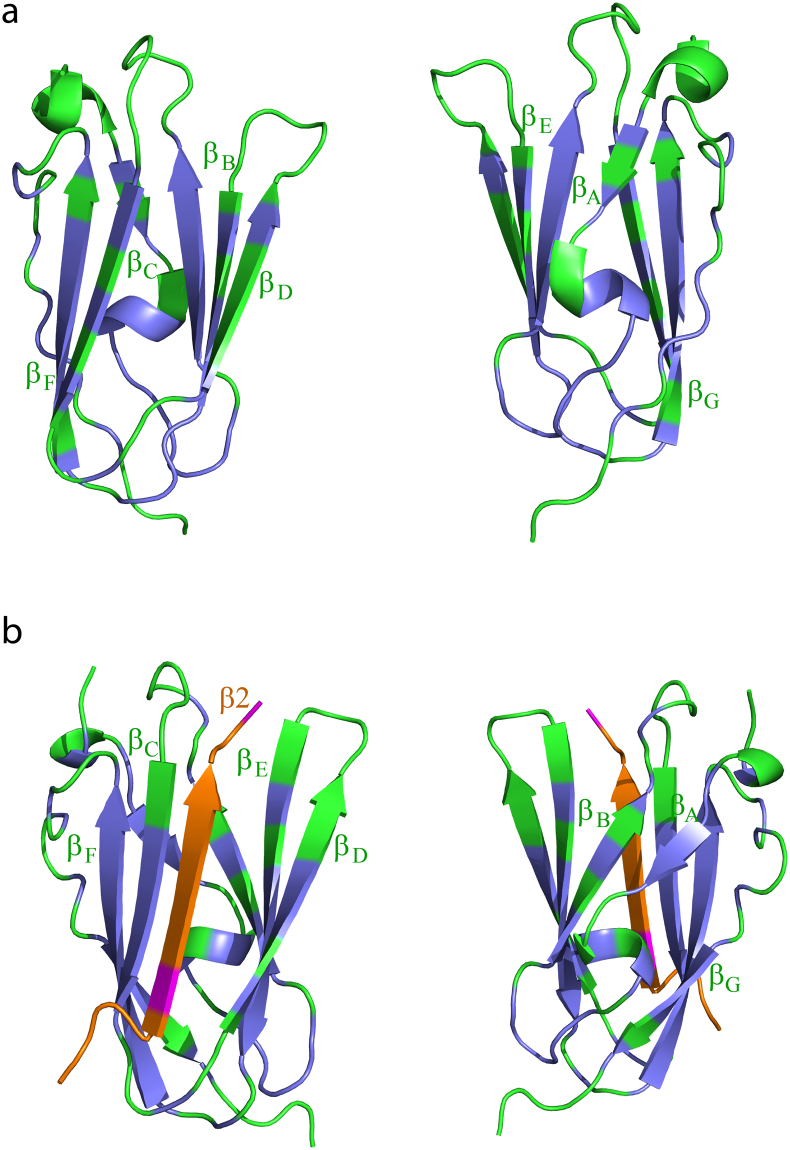
H-D exchange of IgFLNa21 and hybrid IgFLNa21. Ribbon representation of the backbone topology of IgFLNa21 (panel a) and in hybrid IgFLNa21 (panel b) in two orientations. Backbone amide protons showing protection against exchange after 2 hours are highlighted in purple and pink for residues of IgFLNa21 and β2 CT, respectively.

We then compared H-D exchange rates of IgFLNa21 and hybrid IgFLNa21. A series of ^15^N-^1^H HSQC spectra were obtained as a function of time in D_2_O. Residues that were detected in ^15^N-^1^H HSQC spectra after 2 hours of exchange are mapped onto the structures of IgFLNa21 (Fig. 7a) and hybrid IgFLNa21 (Fig. 7b). Amide protons of several residues of both IgFLNa21 and hybrid IgFLNa21 demonstrated slow exchange with solvent, most of these residues are located on the β-strands of the seven stranded β-sheet structure (Fig. 7a and b). However, amide protons from the strands β_C_ and β_D_ of IgFLNa21 were found to be rapidly exchanging with solvent (Fig. 7a). By contrast, more amide protons of the hybrid IgFLNa21 demonstrated slow exchange including residues from strands β_C_ and β_D_ (Fig. 7b). Notably, only two amide protons residues Ser756 and Asn763 of the β2 CT in hybrid IgFLNa21 delineated protection against solvent exchange (Fig. 7b). Amide proton protection characteristics of residues of hybrid IgFLNa21 and IgFLNa21 was further quantified by determining k_ex_ or exchange rate (Table 2). As seen, a number of residues, including amide protons of residues at the strands β_C_ and β_D_ binding pocket, of hybrid IgFLNa21 exhibited a higher k_ex_ or slower exchange kinetics compared to that of IgFLNa21 (Table 2). Notably, residues located in β strands β_A_, β_B_, β_E_, β_F_, far from the β2 CT binding pocket, also delineated slow exchange in hybrid IgFLNa21 (Table 2). Further, it may be noteworthy, k_ex_ of amide protons of residues Ala2238, Vla2241, Ala2243, Arg2263, Val2275, Ser2284, Phe2285, Cys2293, Vla2295, Asp2304, Lys2310 and Ile 2316 can only be determined in the hybrid IgFLNa21 construct (Table 2). Interestingly, few residues, Ala2253, Ser2259, Ala2272, Val2299, Gly2303 and Glu2306 demonstrated somewhat lower k_ex_ in hybrid IgFLNa21 (Table 2). Therefore, the H-D exchange studies demonstrated that there has been a structural stabilization, as evidenced by higher k_ex_ for many residues, of the IgFLNa21domain in complex with β2 CT.

**Table 2:** H-D exchange rate (k_ex_ min^−1^) of the amide protons of hybrid IgFLNa21 and IgFLNa21. Δk_ex_ represents difference of k_ex_ between hybrid IgFLNa21 and IgFLNa21.

Residue	k_ex_ hybrid IgFLNa21	k_ex_ IgFLNa21	Δk_ex_	Structure
Ser756 (β2 CT)	1258.9			β-strand CT
Ans763 (β2 CT)	2119			β-strand CT
Ala2238	784.6	fast exchange		helix
Val2241	26.8	fast exchange		loop
Arg2242	24.7	15.7		loop
Ala2243	3450	fast exchange		βA
Gly2244	312.7	183.3	129.4	βA
Gly2247	2594.5	545.5	2049	helix
Glu2249	862	589.5	316.5	helix
Ala2251	1092	432	660	loop
Glu2252	1349.6	12.5	1337.1	loop
Ala2253	228.6	381.7	−153.1	loop
Gly2254	186	183.6	2.4	loop
Val2255	3865.8	1889	1976.8	loop
Ala2257	1932.4	698.3	1234.1	βB
Glu2258	616.7	335.2	281.5	βB
Ser2259	1049	1279	−230	βB
Trp2261	625.2	204	421.2	βB
Arg2263	2006.4	fast exchange		loop
Ala2272	907.3	1073.8	−166.5	βC
Ala2274	2852.8	1608	1244.8	βC
Val2275	32.8	fast exchange		βC
Glu2276	1910	1143	767	βC
Ser2279	922	88.6	833.4	loop
Glu2282	59	43	16	βD
Ser2284	1061.7	fast exchange		βD
Phe2285	49.7	fast exchange		βD
Cys2293	1476	fast exchange		βE
Val2295	3308.7	fast exchange		βE
Ala2296	2008.5	344	1664.5	βE
Tyr2297	3402.3	1002.9	2399.4	βE
Val2298	2281.4	1371.6	909.8	βE
Val2299	1325	1944	−619	βE
Gly2303	562.4	985	−422.6	loop
Asp2304	2986.5	fast exchange		βF
Tyr2305	2897	1400	1497	βF
Glu2306	1729.4	2513.2	−783.8	βF
Val2307	3621	1377.7	2243.3	βF
Ser2308	1569.5	1226	343.5	βF
Val2309	1164.5	943.6	220.9	βF
Lys2310	1913.8	fast exchange		βF
Phe2311	3133	1021	2112	βF
Glu2314	607.9	354.2	253.7	loop
Ile2316	973	fast exchange		loop
Ser2319	1895	1845	50	loop
Phe2321	1443	647.9	795.1	βF
Val2322	27.3	24.8	2.5	βF
Val2323	5735.3	2507.3	3228	βF
Val2325	1971	1045	926	βF

## Discussion

**Figure 8:**
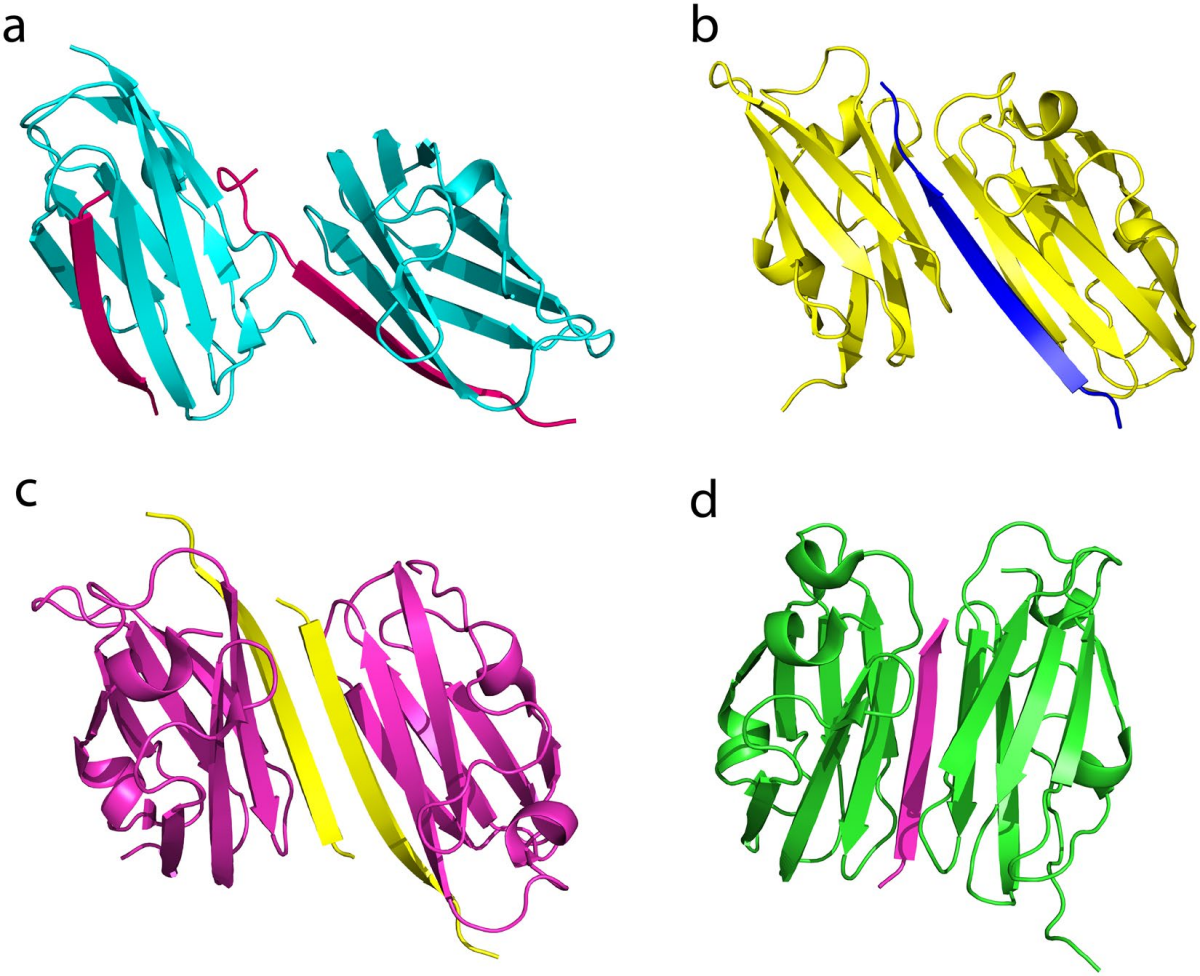
Ribbon representation of the x-ray structures of IgFLNas in complex with peptide ligands. (**a**) Structure of IgFLNa17 in complex with GPIbα peptide. (**b**) Structure of IgFLNa21 in complex with CFTR derived peptide. (**c**) Structure of IgFLNa21 in complex with β7 CT peptide. (**d**) Structure of IgFLNa21 in complex with migfilin derived peptide.

**Figure 9:**
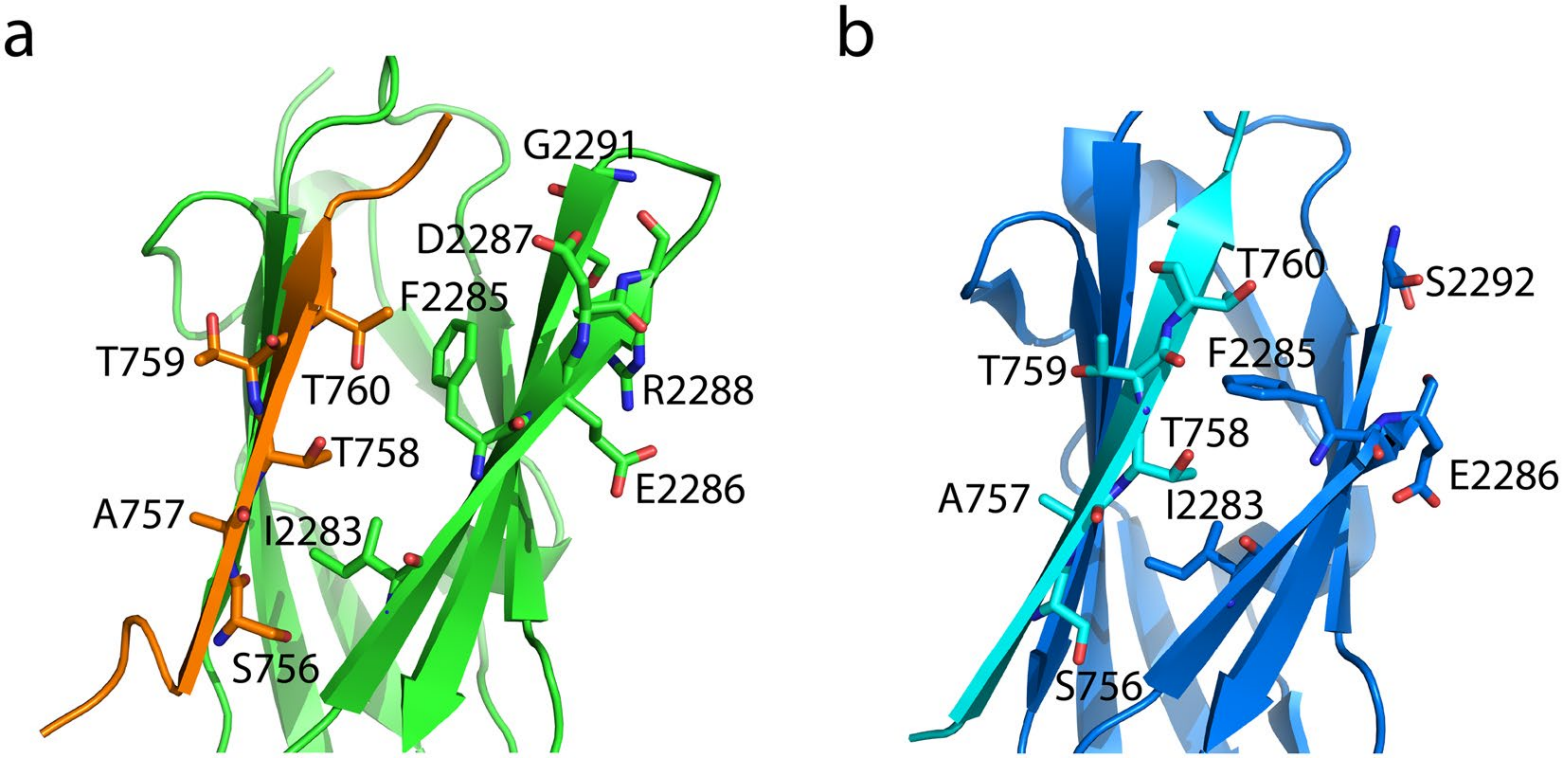
Comparison of x-ray and NMR structures of IgFLNa21-β2CT. An expanded view of the NMR structure (panel a) and x-ray structure (panel b), showing positions of the sidechains of residues at the binding interface. The loop between β strands D/E and residues Asp2287 and Arg2288 deduced in the NMR structure of hybrid IgFLNa21 have been shown.

The IgFLN domains of filamin interact with a large number of proteins, including the β CTs of integrins^31,32^. 3-D structures of IgFLN domains and peptide fragments derived from interacting partners provide important insights into the mode of interactions. Notably, x-ray structures of binary complexes were determined for IgFLNa17/GPIbα peptide^33^, IgFLNa21/β7 CT of integrin^11^, IgFLNa21/CFTR peptide^24^, IgFLNa21/migfilin peptide^34^, IgFLNa21/β2 CT^12^ of integrin. NMR derived solution structure has been reported for the binary complex IgFLNc21/migfilin peptide^13^. More recently, a ternary complex of IgFLNa21/β3 CT/αIIb CT of αIIb/β3 platelet integrin has been solved by solution NMR^20^. The x-ray structure of IgFLNa21/β7 CT shows a dimeric fold mediated by direct interactions between β7 CT peptide between the two subunits (Fig. 8)^11^. Dimeric structures are also determined for IgFLNa21/CFTR^24^, IgFLNa21/migfilin^34^ peptide and IgFLNa17/GPIbα^33^ (Fig. 8). By contrast, x-ray derived structure of IgFLNa21/β2 CT demonstrated a monomeric fold of the binary complex (Fig. 8), and several residues adjoining β-strands β_D_ and β_E_ could not be resolved. In this study, we investigated the structure and dynamics of IgFLNa21 and β2 CT interaction using solution NMR. Due to extensive broadening and weak cross-peaks of ^15^N-^1^H HSQC spectra of IgFLNa21 in the presence of the full-length β2 CT or β2 CT MP, we were unable to resolve the structure of the complex. A similar challenge in resonance broadening was encountered for interaction studies of IgFLNa21 and peptide ligands thereby impeding solution structure analyses^11,33^. To overcome this problem, we generated a hybrid β2CT-IgFLNa21 construct. With this hybrid protein, we obtained high quality NMR spectra that allowed us to determine its 3-D structure and backbone dynamics. The 3-D structure of hybrid IgFLNa21 exhibited a canonical binding mode for β2 CT into the CD face of IgFLNa21 (Fig. 4a and b). The β-strand of β2 CT assumes an anti-parallel orientation with the β-strand C of IgFLNa21, whereby these two β-strands are engaged in potential backbone NH----O=C hydrogen bonding (Fig. 4c). Similar backbone hydrogen bonding appears to be less likely between β-strand D and β2 CT due to farness of the backbone atoms. However, the side chain –OH group of residue Ser756 of β2 CT may form potential hydrogen bond with backbone C=O of residue Ala2281 of the strand β_D_. The cross-strand side chain-side chain packing interactions between the β2 CT and binding pocket β-strands β_C_ and β_D_ are rather limited. Side chains of residues Thr756, Thr758 of β2 CT are in close packing with sidechains of residues Ile2273, Ile2283, Phe2285 of IgFLNa21 (Fig. 4d). In particular, the side chain of Thr756 of β2 CT is centrally located and packed against side chains of Ile2273, Ile2283, Phe2285 (Fig. 4d). Interestingly, a different packing arrangement has been observed in integrin β7CT/IgFLNa complex, whereby side chains of residues Thr783 and Thr785 in β7 CT, equivalent to Thr756 and Thr758 in β2CT, were found to be exposed outside^11^. The x-ray structure of the non-covalent complex of IgFLNa21and β2 CT resembles closely to the solution structure of the hybrid IgFLNa21 (Fig. 9, panels a and b). However, in the x-ray structure, residues, Asp2287-Gly2291, connecting strands β_D_ and β_E_ could not be resolved. Whereas, these residues appeared to be assuming defined conformations or a reverse turn in the hybrid IgFLNa21 structure. Notably, in the tri-peptide segment residues Phe2285-Glu2286-Asp2287, sidechains of residues Phe2285 and Asp2287 point toward the binding pocket and in proximity to the β2 CT peptide (Fig. 9a). High S^2^ values were estimated for some of the residues in hybrid filamin e.g. Glu2286, Asp2287, Lys2261 and Asp2262 in the reverse turn, indicating limited backbone motion (Fig. 6a). Heteronuclear NOE and high S^2^ values >0.8 demonstrated that most of the residues in the β-sheet structures of hybrid IgFLNa and IgFLNa21 are motionally rigid at ps-ns time scale of motion. Residues Ser756, Ala757, Thr758, Thr759 and Asn763 of β2 CT, in hybrid IgFLNa21, are found to be delineating backbone motional rigidity (Fig. 6a). Analyses of the dynamics of interactions further revealed that the insertion of β2 CT into hybrid IgFLNa21 has prompted conformational exchange processes for certain residues at the binding pocket. Whereby higher R_ex_ values of residue Thr759 of β2 CT and residues Ala2272, Ile2273, Ala2281 and Glu2316 of IgFLNa21 in hybrid filamin were estimated (Fig. 6b). Importantly, these residues of IgFLNa21 are in the proximity of the β2 CT. In addition, residue Thr759 of β2 CT is in close proximity with residues Ala2272 and Ile2237, whereas, backbone carbonyl of residue Ala2281 may form potential hydrogen bond with the sidechain of residue Ser756. In order to understand conformational stability, H-D exchange rate (k_ex_) was determined for hybrid IgFLNa21 and wild-type IgFLNa21. Higher k_ex_ rate observed for several residues of hybrid IgFLNa21 indicated a conformational stabilization upon insertion of β2 CT (Fig. 7, Table 2). By contrast, most of the amide protons, except residues Ser756 and Asn763, of β2 CT in hybrid IgFLNa21 experienced a facile H-D exchange, suggesting a lower structural stability of the β2 CT in the hybrid IgFLNa21 (Fig. 7).

Our data provide important molecular insights into β2 CT and IgFLNa21 interaction. Based on the current model, filamin binds to integrin β CT and occludes the membrane distal (MD) binding region of the activating protein talin^10–12^. Two mechanisms that could lead to the dissociation of filamin from the integrin β CT are: kindlin-migfilin-mediated displacement and phosphorylation of the TTT-motif in the ββ CT^12,29,30^. The first mechanism involves the binding of kindlins to the membrane distal NxxY/F motif in the integrin β CT. Kindlins interact with migfilin, and the migfilin binding site on filamin overlaps with that of integrin β2 and β7 CTs^13,35^. Hence, kindlin-migfilin complex binding to the integrin β CT displaces the bound filamin, leading to integrin activation^13,35^. The second mechanism involves phosphorylation of the integrin β CT. It has been shown that phosphorylation of Thr758 in the TTT-motif of the β2 CT not only disrupts filamin binding, it promotes the association of scaffold protein 14-3-3ζ that is a positive regulator of integrin activation^12^. Our recent study also demonstrated that pThr758-β2 CT can simultaneously interact with 14-3-3ζ and talin^36^ .The TTT-motif is highly conserved in the integrin β2, β3, β5, β6 and β7 CTs^5^. Whether the motif is amenable to phosphorylation in all these CTs requires further studies. In the case of β2 CT, in which Thr758 of the TTT-motif can be phosphorylated, the kinase involved has not been identified. Based on the x-ray structure of IgFLNa21 in complex with the β2 CT complex^12^, Thr758 is involved in hydrophobic packing interactions with residues Ile2283 and Phe2285 of IgFLNa21^12^. We also observed the same mode of packing in this study. A similar hydrophobic packing was reported for the TST-motif in integrin β3 CT when in complex with IgFLNa21^17^ .Conceivably, this packing arrangement would limit the accessibility of Thr758 in the β2 CT to phosphorylating kinase(s), thereby preventing the activation of β2 integrins. However, this would also imply that the dissociation of filamin from the β2 CT as a consequence of Thr758 phosphorylation is improbable unless there is some form of dynamic instability in the β2 CT/IgFLNa21 complex. Our data demonstrated high S^2^ values of residues of β2 CT indicated rigidity of N-H bond motions at ps-ns time scale, however, limited protection of amide protons of most of the residues of β2 CT indicated existence of potential local structural fluctuations that would result a facile H-D exchange process^37^. Local structural fluctuations have been described as a significant mode of amide proton exchange with solvent in well folded proteins in the absence of any denaturant^37^. Further, higher R_ex_ of interfacial residues Thr759 of β2-CT and Ala2272 and Ile2237 of IgFLNa21 demonstrated β2-CT binding perhaps promoted conformational exchange processes. Taken together, we surmise that existence of multiple conformational states of β2-CT and IgFLNa21 would likely to disfavor high affinity binding interactions. Most importantly, the augmented dynamical characteristics and low conformational stability of β2 CT in complex with IgFLNa21 would favor the phosphoryalation of the residue T758 during the course of activation of integrins. In conclusion, transient protein-ligand interactions in proteomes are prevalent, however determination of atomic-resolution structures and dynamics in solution is highly challenging. Our study implicated that covalent tethering of transiently interacting protein domain, IgFLNa21 and binding ligand, β2-CT could be an important strategy for elucidation of 3-D structure and residue specific dynamical analyses. Further, our study have provided important molecular insights towards filamin mediated regulation of integrins activation.

## Materials and Methods

### Synthetic peptides

Synthetic integrin full-length β2-CT (residues 724–769) and β2-CT MP (K^724^ALIHLSDLREYRRFEKEKLKSQWNND^750^) peptide were purchased from GLBioChem™ (Shanghai, China) and were further purified using reverse phase HPLC (Waters™ 2489). The mass of the peptides was confirmed by mass spectrometry.

### Protein expression and purification

Synthetic genes, with optimized *E. coli* codons, of IgFLNa21 domain (Gly^2236^-Ser^2329^) and hybrid IgFLNa21 domain (P^752^LFKSATTTVMN^763^-GASGSGASGS-G^2236^-S^2329^ were sub cloned into pET24a and pET14b vectors (Novagene), respectively. Transformed *E. coli* rosetta cells (Merck) were grown at 37 °C in rich media (LB) or supplemented M9 minimal media containing either ^15^N ammonium chloride and/or ^13^C glucose (Cambridge Isotope Laboratories). Protein production was induced by addition of 0.5 mM IPTG at OD_600_ ~ 0.6–0.7 and cells were further grown at 20 °C for 10–12 hours. Cells were harvested through centrifugation and pellets were resuspended in buffer A (50 mM sodium phosphate buffer and 300 mM NaCl, pH 8.0) followed by cell lysis using sonication. Lysates were loaded onto Ni-NTA column (GE) for His-tag based affinity purification using AKTA FPLC UPC-900 system (GE Healthcare UK Ltd., England). His-tagged proteins were first eluted using buffer B (50 mM sodium phosphate buffer, 500 mM imidazole, and 300 mM NaCl, pH 8.0), then exchanged into buffer C (20 mM sodium phosphate buffer and 50 mM NaCl, pH 6.0). Protein samples were further purified by size-exclusion chromatography in buffer C using a Superdex 200 10/300 column with a flow rate of 0.3 ml/min. NMR samples were prepared, through buffer exchange, in buffer D (25 mM sodium phosphate buffer, 5 mM NaCl, 2 mM DTT and 10% D_2_O, pH 6.5).

### NMR experiments

NMR experiments were carried out at 298 K on a Bruker DRX 600 MHz spectrometer equipped with cryoprobe. Data were acquired and processed using Bruker Topspin 3.0 software and analyzed by Sparky (T.D. Goddard and D.G. Kneller, University of California, San Francisco). For hybrid IgFLNa21, concentration 300 μM, a suit of three dimensional heteronuclear NMR experiments, HNCACB and CBCA(CO)NH, ^15^N-TOCSY-HSQC (mixing time: 80 ms),^13^C-NOESY-HSQC (mixing time: 120 ms) and ^15^N-NOESY-HSQC (mixing time: 120 ms) were carried out for backbone and sidechain resonance assignments and NOE analyses. ^15^N-^1^H HSQC spectrum of IgFLNa21 were assigned by acquiring HNCACB and CBCA(CO)NH spectra. All NMR spectra were referenced to DSS. Interaction studies were performed by acquiring ^15^N-^1^H HSQC spectra of IgFLNa21, 100 μM concentration, either with sequential additions of full-length β2-CT or β2-CT MP peptide at 1:0, 1:1, 1:2 and 1:3 ratios. ^15^N-labelled IgFLNa21 and hybrid IgFLNa21 were lyophilized from buffer D and re-suspended in 100% D_2_O for H-D exchange experiments. A series of ^1^H-^15^N HSQC spectra were obtained for both proteins with 15 to 30 minutes interval. Hydrogen exchange rate k_ex_ were calculated using a mono-exponential decay equation.

### Structure calculations

Hybrid IgFLNa21 protein backbone φ and ψ torsion angles were predicted using TALOS+ server. Crosspeaks from ^13^C-HSQC-NOESY and ^15^N -HSQC-NOESY, were selected and integrated using SPARKY. Distance constraint and structure calculation were calculated using CYANA 2.1 automated NOE assignment protocol. Structures were further refined by X-plore-NIH using explicit water. A final ensemble of 20 structures with low RMSD and target function were submitted to BMRB. PSVS was used for validation of NMR derived structure quality.

### ^15^N relaxation measurement and model-free analysis

Standard NMR pulse programs were used to obtain the ^15^N longitudinal relaxation rate (R_1_ = 1/T_1_), ^15^N transverse relaxation rate (R_2_ = 1/T_2_) and ^15^N steady state heteronuclear NOE for IgFLNa21 and hybrid FLNa21 at 298 K using cryoprobe equipped Bruker DRX 600 MHz NMR. A series of ^1^H-^15^N HSQC experiments were acquired for both ^15^N-labelled IgFLNa21 and hybrid IgFLNa21 to obtain T_1_ and T_2_ relaxation rates as a function of delays. The T_1_ and T_2_ relaxation rates were obtained by fitting spectral peaks height to a mono-exponential decay function: PI = C*exp(−t/T) where PI, C, T, and t represent peak intensity, constant, relaxation rate constant, and experimental time point, respectively. The ^15^N heteronuclear NOE experiments were conducted in the presence or absence of proton saturation with the inter scan delay set to 3 s. Steady state NOE value for each ^15^N nucleus was calculated as a ratio of cross peak heights of the spectra collected with proton saturation and without any proton saturation. R_1_, R_2_ and steady state NOE calculations were carried out using NMR software *relax*. Model free analyses were performed using FASTModelFree and ModelFree4 software packages.

### Availability of materials and data

All data generated or analyzed during this study are included in this published article (and its Supplementary Information files).
